# Organ-specific SPECT activity calibration using 3D printed phantoms for molecular radiotherapy dosimetry

**DOI:** 10.1186/s40658-016-0148-1

**Published:** 2016-07-13

**Authors:** Andrew P. Robinson, Jill Tipping, David M. Cullen, David Hamilton, Richard Brown, Alex Flynn, Christopher Oldfield, Emma Page, Emlyn Price, Andrew Smith, Richard Snee

**Affiliations:** Schuster Laboratory, School of Physics and Astronomy, The University of Manchester, Manchester, M13 9PL UK; Christie Medical Physics and Engineering (CMPE), The Christie NHS Foundation Trust, Wilmslow Road, Manchester, M20 4BX UK

**Keywords:** Activity quantification, SPECT, Molecular radiotherapy, 3D printing, Absorbed dose

## Abstract

**Background:**

Patient-specific absorbed dose calculations for molecular radiotherapy require accurate activity quantification. This is commonly derived from Single-Photon Emission Computed Tomography (SPECT) imaging using a calibration factor relating detected counts to known activity in a phantom insert.

**Methods:**

A series of phantom inserts, based on the mathematical models underlying many clinical dosimetry calculations, have been produced using 3D printing techniques. SPECT/CT data for the phantom inserts has been used to calculate new organ-specific calibration factors for ^99*m*^Tc and ^177^Lu. The measured calibration factors are compared to predicted values from calculations using a Gaussian kernel.

**Results:**

Measured SPECT calibration factors for 3D printed organs display a clear dependence on organ shape for ^99*m*^Tc and ^177^Lu. The observed variation in calibration factor is reproduced using Gaussian kernel-based calculation over two orders of magnitude change in insert volume for ^99*m*^Tc and ^177^Lu. These new organ-specific calibration factors show a 24, 11 and 8 % reduction in absorbed dose for the liver, spleen and kidneys, respectively.

**Conclusions:**

Non-spherical calibration factors from 3D printed phantom inserts can significantly improve the accuracy of whole organ activity quantification for molecular radiotherapy, providing a crucial step towards individualised activity quantification and patient-specific dosimetry. 3D printed inserts are found to provide a cost effective and efficient way for clinical centres to access more realistic phantom data.

## Background

Accurate activity quantification is essential for patient-specific absorbed dose calculations for Molecular Radiotherapy (MRT). Current activity quantification for Single-Photon Emission Computed Tomography (SPECT), as commonly used in MRT, often relies on camera-specific calibration factors, determined from phantom data. These camera-specific calibrations are used to compensate for the many quantitative inaccuracies in SPECT imaging [1]. Calibration factors determined from physical phantom data are highly dependent on the specific geometry and activity distribution being considered. The geometry of phantom inserts used for SPECT activity calibration is often far removed from both the clinical situation and the mathematical models which provide the basis of the most common MRT dosimetry calculations. The OLINDA/EXM software [2] applies the MIRD schema for whole organ dosimetry [3], using mathematical models defined in [4–7], and is currently the only FDA-approved general MRT dosimetry code. Whilst these generic organ models do not reflect the range of MRT patient morphologies, they do allow dose calculations to be adapted to consider individual organ masses and patient age and sex. As such, the ready availability of phantoms providing activity distributions which better approximate clinical MRT distributions will provide a significant step forward towards accurate patient-specific dosimetry for all clinical centres performing MRT.

In this work, a series of novel phantom inserts corresponding to organ models used in common MRT dosimetry systems have been produced, based on the models described in [4]. The inserts have been printed using fused filament modelling (3D printing) with acrylonitrile butadiene styrene (ABS) plastic. This paper presents SPECT/CT data for the printed inserts containing ^99*m*^Tc and ^177^Lu in a water-filled phantom. The suitability of ABS plastic as a tissue analogue for MRT phantom production is demonstrated for the first time. SPECT calibration factors for ^99*m*^Tc and ^177^Lu, corresponding to the MIRD organ geometries defined in [4], are determined. A significant dependence on organ shape for calibration factors is demonstrated and compared to calculated values. Finally, the influence of improved organ-specific calibration factors on clinically representative absorbed dose calculations (using the OLINDA/EXM package) is evaluated. 3D printing production allows the phantom inserts to be made available [8] in stereolithography (STL) format, which can be printed using a commercial 3D printer or printing service. Providing access to the phantom inserts described in this work will allow any clinical centre to accurately calibrate clinical SPECT activity quantification for input to whole organ MRT dosimetry calculations, resulting in improved dose calculations.

## Methods

### 3D printed phantom inserts

**Fig. 1:**
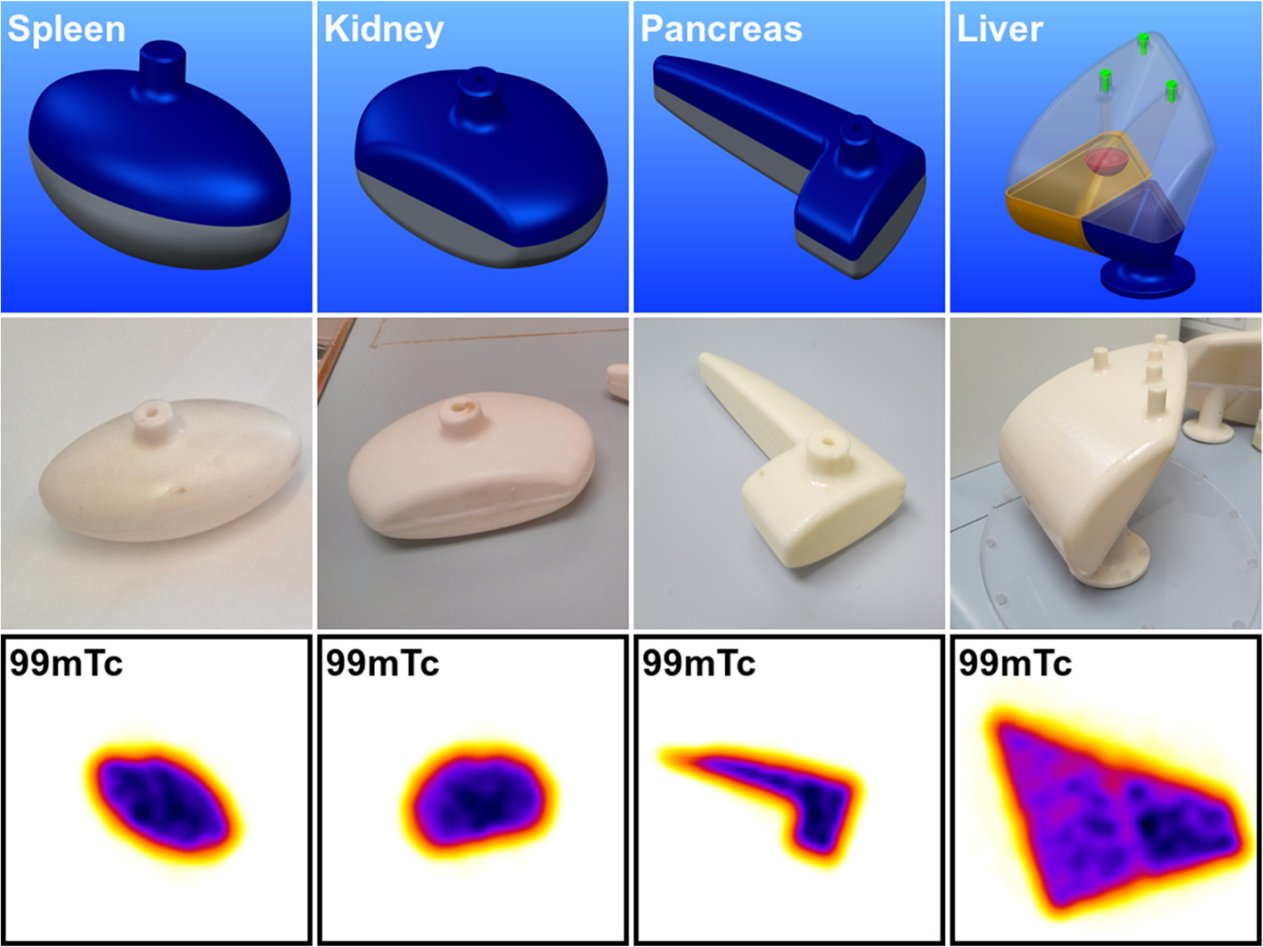
CAD models designed from adult male mathematical models defined in [4] (*top*). The liver model (*right*) has been extended to include a spherical tumour and separate lobes. Organ inserts printed with white ABSplus (*middle*). Central slice through SPECT reconstruction of inserts containing ^99*m*^Tc (*bottom*)

The designs and final printed inserts are shown in Fig. 1. The inserts consist of shells with 2-mm thick walls (3 mm for the larger liver model) which can be filled with a radioisotope solution. The inserts have integrated attachment and filling ports which can be fitted with standard M5 screw threads, allowing them to be used in a variety of commercial phantoms. Inserts were designed based on the adult male models for MRT dose-limiting organs as described in [4] (see Table 1 for details). Additional inserts corresponding to age-5 and age-10 kidneys were also produced. The adult male liver model has been extended to include two separate lobes and a small (16 ml) tumour. The variation of the insert volumes from those described in [4] (shown in Table 1) reflects the physical constraints of the 3D printing technique. Binary STL files of the models, compatible with most common models of printer, are available online [8]. The inserts used in this work were printed from ABS plastic (ABSplus - Stratasys Ltd) with a 0.178-mm layer thickness (solid wall interior) using a Dimension Elite printer (Stratasys Ltd).

**Table 1:** Details of phantom inserts, mathematical models volumes [4] and radioisotope activities

Organ ^a^	Model volume ^b^	Printed insert volume	Activity ^99*m*^Tc	Activity ^177^Lu
	(cm ^3^)	(cm ^3^)	(MBq)	(MBq)
Spleen	176.0	195.26 ± 0.02	122.3 ± 0.3	639.1 ± 1.2
Pancreas	83.8	103.26 ± 0.02	110.4 ± 0.3	397.4 ± 0.7
Liver	1830.0	1577.49 ± 0.02	515.3 ± 1.2	2976.8 ± 5.6
Liver (left) ^c^	–	479.00 ± 0.02	172.4 ± 0.8	924.2 ± 4.0
Liver (right) ^c^	–	1098.49 ± 0.02	342.9 ± 0.9	2052.6 ± 3.9
Liver (tumour) ^c^	–	16.49 ± 0.02	6.4 ± 0.2	43.0 ± 0.5
Kidney	144.0	169.81 ± 0.02	125.0 ± 0.3	1024.6 ± 1.2
Kidney age 10	80.3	99.54 ± 0.02	92.5 ± 0.2	589.5 ± 0.2
Kidney age 5	55.5	64.96 ± 0.02	32.6 ± 0.1	383.9 ± 0.2

^a^Adult male organs unless specified

^b^Values from [4]

^c^Additional sub compartment added to model

### SPECT phantom data

The phantom inserts were filled with saline containing either ^99*m*^Tc pertechnetate or ^177^Lu-Dotatate (with the activities given in Table 1) and placed individually in the centre of a water-filled elliptical phantom without activity (9.5 l, 186-mm high with 305 and 221 mm radii). The phantom was imaged in the same central position on the bed with a fixed head radius of 25 cm for each insert to ensure a constant SPECT resolution. Positioning both the phantom and insert centre-of-mass in the centre of the SPECT field-of-view ensures optimal performance of CT-based attenuation correction for SPECT reconstruction [9]. SPECT/CT data were acquired for each insert and radioisotope using an Infinia-Hawkeye-4 SPECT/CT camera (GE Healthcare). LEHR and MEGP collimators were used for ^99*m*^Tc and ^177^Lu, respectively. Scans were performed using 60 views in a step-and-shoot circular orbit (20 s per view for ^99*m*^Tc and 40 s per view for ^177^Lu). Projection data was acquired in a matrix of 128 × 128 4.42-mm pixels for the 140 ±10*%* keV photopeak of ^99*m*^Tc and the 208 ±10*%* of ^177^Lu. Data was also acquired for energy windows adjacent to the photopeak (^99*m*^Tc 120 ±3*%* keV, ^177^Lu 181 ±3*%* and 236 ±3*%* keV) allowing dual or triple energy windows scatter correction to be applied [10]. The phantom data were reconstructed using a clinical OSEM algorithm (GE OSEM) with ten subsets and four iterations. This reconstruction algorithm does not include collimator-detector response (CDR) compensation, which can improve spatial resolution and reduce spill-out of counts. It should be noted however that CDR compensation may create ringing artefacts which change the distribution of counts within a volume of interest (VOI) [11]. The reconstruction parameters were found to provide stable quantification for all organ VOIs for both ^99*m*^Tc and ^177^Lu, with the total number of reconstructed counts increasing by <0.7 *%* for higher numbers of reconstruction iterations. Attenuation correction (AC) was applied using Hawkeye CT data (140 kV, 2.5 mA) acquired as part of the scan protocol. The central slice through the SPECT reconstructions of the inserts, demonstrating the activity distribution, is shown in Fig. 1.

### Organ-specific SPECT calibration factors

Generally, a calibration factor, cf, is defined which relates the count rate (counts per second, cps) in a reconstructed SPECT image volume-of-interest (VOI) to the total activity (MBq) in the VOI in order to provide activity quantification of SPECT imaging for MRT patients,

1 $$  \mathrm{cf = \frac{counts}{total~activity \cdot time} = \frac{count~rate}{total~activity}}.  $$

A VOI defining each organ insert was manually outlined using the Hawkeye CT data and transferred to the SPECT image. A corresponding calibration factor (*cf*_*voi*_) for ^99*m*^Tc and ^177^Lu was calculated for each VOI. The total number of isotropic voxels, of size 4.42 mm (as used for the SPECT images), defining the inner volume for each VOI was also measured. The sensitivity of a SPECT camera is system specific. Experimentally determined SPECT calibration factors are highly dependent on the choice of reconstruction parameters, including scatter correction, attenuation correction and partial volume corrections. In addition, when applying any phantom-based SPECT calibration factors to patient data, the activity concentration outside the VOI, which varies for each patient and organ, must also be accounted for. In order to isolate the influence of VOI shape on calibration factor from these effects, identical reconstruction parameters and phantom position were used for all scans with no background activity. This ensures that any variation in measured calibration factors are due to the choice of phantom insert alone, highlighting the benefits of using more representative organ geometries.

#### Measuring true camera sensitivity calibration factor (***cf***_***true***_)

The true camera sensitivity calibration factor, c*f*_*true*_, gives the SPECT camera sensitivity (cps/MBq) when no partial volume effects are present. To determine c*f*_*true*_, additional SPECT/CT data was acquired for a 7.3-l cylindrical phantom (height 200 mm and radius 108 mm) containing 712(1) MBq and 3551(7) MBq of ^99*m*^Tc and ^177^Lu, respectively. The phantom was imaged using the same acquisition and reconstruction protocols previously described for the phantom inserts. The true calibration factor was calculated (equation 1) from the uniform phantom data using the reconstructed total number of counts in the SPECT field of view.

#### VOI calibration factor

The finite resolution of a reconstructed SPECT image causes the measured calibration factor for a specific VOI (c*f*_*voi*_) to deviate from the “true” camera sensitivity calibration factor, c*f*_*true*_ due to spatial blurring of the count distribution. The measured counts detected within a VOI (count*s*_*voi*_) can therefore be defined by,

2 $$  \mathrm{counts_{voi} = counts_{true} - counts_{outside}}  $$

where count*s*_*true*_ is the “true” number of counts in the VOI and count*s*_*outside*_ represents counts originating from the VOI which are detected outside the VOI. For a simple geometry, described by a known mathematical function, the effects of finite spatial resolution and discritization of counts in a voxelised VOI may be considered individually. However, obtaining an analytical solution for spatial blurring of an arbitrarily shaped volume is often not possible. In this work, a voxelised approach, which can be applied to any VOI and combines the effect of discretization and limited spatial resolution, is presented. For a VOI with nVoxels, total voxels count*s*_*outside*_ can be defined as,

3 $$  \mathrm{counts_{outside}} = \sum_{i=1}^{\mathrm{{nVoxels}}}\alpha_{i}c_{i}  $$

where *α*_*i*_ is the fraction of true events in voxel *i* which are located outside of the VOI and *c*_*i*_ is the number of true events in the voxel. The value of *α*_*i*_ can be calculated for an individual voxel within a VOI, centred at *x*_*i*_,*y*_*i*_ and *z*_*i*_, by considering the convolution with a 3D Gaussian kernel, *g*(*x*,*y*,*z*,*σ*), integrated over the volume of the VOI.

4 $$  \alpha_{i} = 1-\int_{z_{a}-z_{i}}^{z_{b}-z_{i}}\int_{y_{a}-y_{i}}^{y_{b}-y_{i}}\int_{x_{a}-x_{i}}^{x_{b}-x_{i}} g(x_{i},y_{i},z_{i},\sigma)dx\,dy\,dz  $$

where *x*_*a*_,*x*_*b*_,*y*_*a*_,*y*_*b*_,*z*_*a*_ and *z*_*b*_ are the closest perpendicular distances to the VOI boundary (for a voxel centred at *x*_*i*_,*y*_*i*_ and *z*_*i*_) in the *x*, *y* and *z* dimensions, respectively. *g*(*x*,*y*,*z*,*σ*) is defined as,

5 $$  g(x,y,z,\sigma) = \frac{1}{\sigma^{3}(2\pi)^{\frac{3}{2}}}e^{-\left(\frac{x^{2}+y^{2}+z^{2}}{2\sigma^{2}}\right)}  $$

and *σ* is related to the full width at half maximum (FWHM) of the SPECT camera,

6 $$  \sigma(\text{FWHM}) = \frac{\text{FWHM}}{2\sqrt{2\,ln2}}.  $$

In the case of a phantom insert with a uniform activity distribution (constant *c*_*i*_), combining Eqs. 1–3 gives the variation in measured calibration factor (c*f*_*voi*_) from (c*f*_*true*_) for a specific VOI, with *α*_*i*_ defined by Eq. 4,

7 $$  \frac{\mathrm{cf_{voi}}}{\mathrm{cf_{true}}} = 1 - \frac{\sum\limits_{i=1}^{\text{nVoxels}}\alpha_{i}}{\text{nVoxels}}.  $$

The value of *α*_*i*_, and therefore c*f*_*voi*_, is highly dependent on both the specific three-dimensional geometry of a VOI and the resolution of the SPECT camera. In addition, for voxels located on the edge of a VOI, the voxel size will define the closest perpendicular distance to the VOI boundary. In general, the value of *α*_*i*_ for a voxel on the perimeter of a VOI will vary significantly according to position on the surface, whilst for centrally located voxels, *α*_*i*_∼0 (when the VOI is sufficiently large compared to FWHM).

For example, with a spatial resolution of 10 mm, an isotropic 4.42-mm voxel size and no activity outside the VOI, Eq. 4 predicts *α*_*i*_ = 0.30 (a 30 % loss in counts) for a voxel in the centre of a face of a cubic VOI of length 300 mm. For a voxel on the centre of one edge or corner of the VOI perimeter, *α*_*i*_ = 0.51 (51 % loss) and 0.66 (66 % loss of counts), respectively, and for a single cubic voxel, *α*_*i*_ = 0.94. The corresponding values of *α*_*i*_ for an infinitesimal voxel size (point source) are 0.5 (centre of a face), 0.75 (edge of cube) and 0.875 (corner). In general, for a voxel on the edge of a VOI, the value of *α*_*i*_ can be expressed as *α*_*i*_(*x*,*σ*)^*n*^ where *n* is the number of VOI edges intersected by the voxel. The spill-out fraction from a VOI can be approximated as being proportional to are*a*_*voi*_/volum*e*_*voi*_.

When activity is present outside the VOI, then Eq. 7 has an additional spill-in component, which is analogous to Eq. 3 but summed over all voxels containing activity outside the VOI, increasing the value of c*f*_*voi*_/c*f*_*true*_.

### Absorbed dose calculations

Absorbed dose calculations were performed on VOIs corresponding to the liver, spleen and kidneys for co-registered sequential SPECT/CT images of a patient receiving ^177^Lu-Dotatate therapy with the OLINDA/EXM package [2]. VOIs for these organs were manually defined at each imaging time point on the CT images fused with the SPECT data. The patient CT images showed no evidence of motion between SPECT and CT acquisition or imaging artefacts at the organ edges which may affect VOI definition. Separate time-activity curves (for imaging times, 1-, 24-, 71- and 143-hr post injection) were generated, using a calibration factor for each organ, or with standard sphere (113 ml, 27-mm radius) and cylinder calibration factors (278 ml, 44-mm diameter and 183-mm height). The residence time (*A*_cumulative_/*A*_injected_), as defined by [2], for each organ was calculated using a fit to a single exponential decay and an initial injection activity of 7448 MBq. MRT time-activity data is highly patient-specific and varies significantly for different organs and tumour burdens, often requiring detailed pharmacokinetic modelling [12]. For the patient time-activity data chosen in this study, the difference in calculated residence time for a bi-exponential fit, compared to a single exponential, was found to be <1 %, significantly smaller than the uncertainty in *A*_cumulative_ (∼12 %). Masses were calculated from CT imaging for the liver, spleen and kidneys; otherwise, organ masses were scaled to the total body weight of the patient.

## Results

### ABS material properties

CT data from SPECT/CT scans of the phantom inserts were first used to measure the CT attenuation of the ABS material. The ABS material in the inserts was found to have a mean value of −54±13 HU. The effective density of an ABS print depends on the model geometry and properties of the printer. The inserts produced in this work have a mean effective density of 1.01 ±0.02 g/cm ^3^, corresponding to a packing density of 0.97 ±0.02. ABS printed material was found to provide a closer analogue to water (−7.4±8.7 HU) than perspex (116 ±9 HU) reducing the artificial boundary effect between regions of activity in the phantom. This result demonstrates that ABS printing, the most commonly available and affordable form of 3D printing, is a suitable technique for MRT phantom production.

### Whole organ calibration factors

**Fig. 2:**
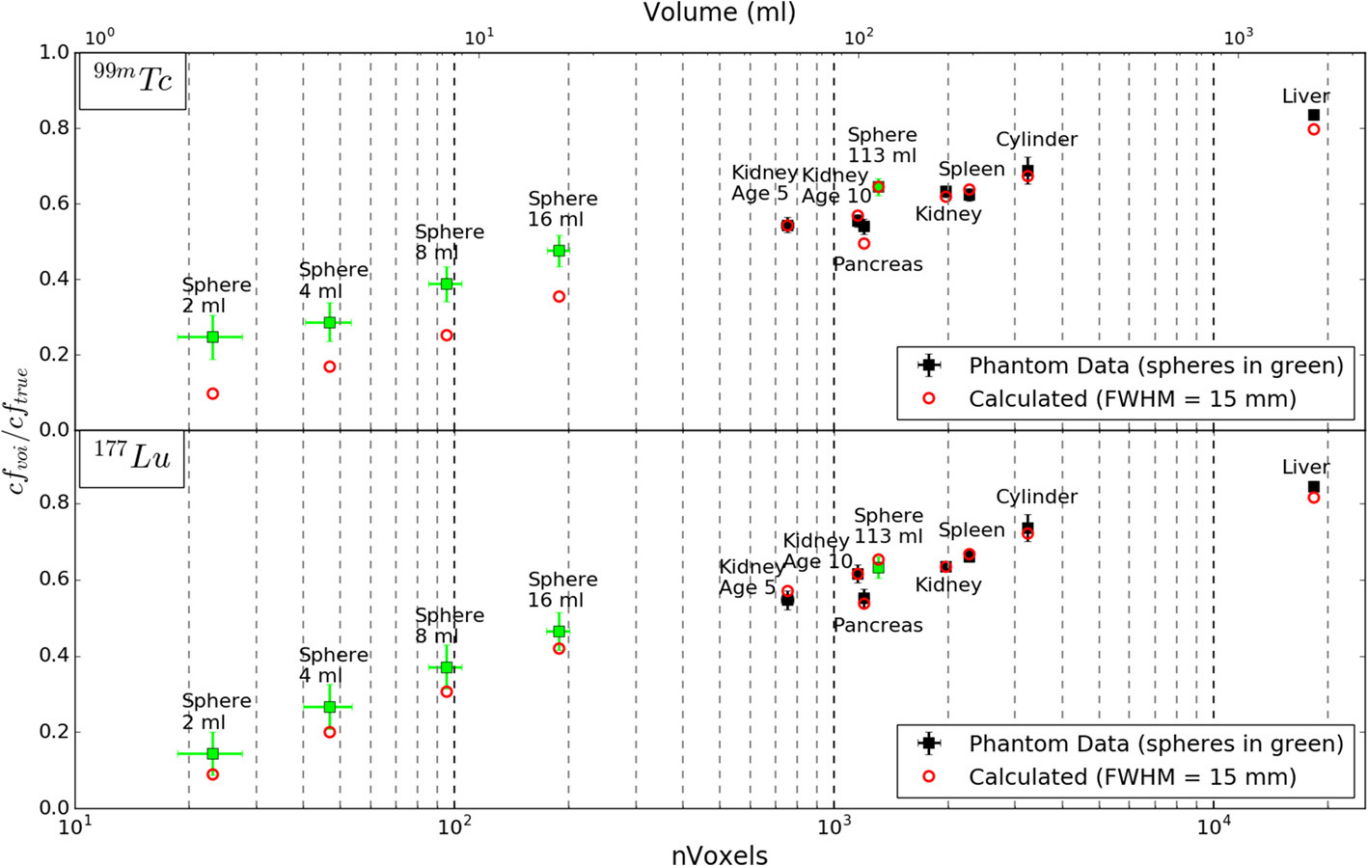
Ratio of VOI calibration factor (c*f*_*voi*_) to true camera sensitivity calibration factor (c*f*_*true*_), as a function of number of voxels in VOI (nVoxels). Data is shown for phantom measurements (*black*and *green squares*) and corresponding calculated values (*red circles*) (Eq. 7). The plotted standard uncertainties include a ±1 voxel (4.42 mm) uncertainty in positioning the VOIs. Note logarithmic scale on x-axis

The measured true camera sensitivity calibration factors (c*f*_*true*_) for ^99*m*^Tc and ^177^Lu are given in Table 2. These values were calculated from uniform phantom data using the reconstructed total number of counts in the SPECT field of view. The ratio of the measured calibration factor (c*f*_*voi*_) to c*f*_*true*_ for ^99*m*^Tc and ^177^Lu, calculated using CT-defined VOIs outlining the phantom inserts, is shown in Fig. 2 (black squares). Data is presented for non-spherical inserts consisting of 3D printed organ inserts, for a 278-ml perspex cylinder and for a series of spherical perspex inserts (2 to 113 ml). The plotted standard uncertainties include a ±1 voxel (4.42 mm) uncertainty in positioning the VOIs.

**Table 2:** True camera sensitivity calibration factor (c*f*_*true*_) determined from reconstructed total counts in field of view for a 7.3-l cylindrical phantom containing uniform activity

Isotope	Phantom activity	c*f*_*true*_
	(MBq)	(cps/MBq)
^99*m*^Tc	712 ± 1	150.49 ± 0.34
^177^Lu	3551 ± 7	11.74 ± 0.02

As predicted (by Eqs. 4 and 7), the value of c*f*_*voi*_/c*f*_*true*_ approaches 1 for the larger organ inserts and is significantly reduced for the smaller spherical inserts. The theoretical value of c*f*_*voi*_/c*f*_*true*_ for a uniform activity distribution, calculated using Eq. 7 and a FWHM of 15 mm, is shown for each VOI in Fig. 2 (red circles). There is a good agreement with the measured values of c*f*_*voi*_/c*f*_*true*_ for the larger inserts, including the 113-ml sphere. However, the measured values of c*f*_*voi*_/c*f*_*true*_ is systemically larger for the smaller spherical inserts (2–16 ml). In this paper, data is presented without scatter correction to ensure the influence of organ shape is isolated. It should be noted however that the same tendency in c*f*_*voi*_ (with a corresponding reduction in counts per MBq) is observed for both ^99*m*^Tc and ^177^Lu when consistent scatter correction is applied to these data (using the previously defined scatter windows).

### Absorbed dose calculations

Table 3 shows ^177^Lu cumulative activity and absorbed dose calculations for the liver, spleen and kidneys using organ-specific activity calibration factors from 3D inserts (as previously described). Values are also given for a standard spherical insert (113 ml) and cylindrical insert (278 ml) calibration factor for comparison, which approximate the average organ volumes in Table 1 and are commonly available to clinical centres.

**Table 3:** Clinically representative ^177^Lu MRT absorbed dose calculation using calibration factors from 3D printed organ inserts and spherical and cylindrical inserts

	Organ factor		Sphere factor ^a^			Cylinder factor ^b^		
	*A*_cumulative_	Abs. Dose	*A*_cumulative_	Abs. dose	*Δ* *Dose* ^c^	*A*_cumulative_	Abs. dose	*Δ* *Dose* ^c^
	(GBq.Hrs)	(mGy)	(GBq.Hrs)	(mGy)	(%)	(GBq.Hrs)	(mGy)	(%)
Spleen	48.8 ± 9.2	10500	54.9 ± 10.1	11800	11.0	43.4 ± 0.8	9370	−12.1
Liver	59.8 ± 4.2	2690	78.6 ± 5.5	3540	24.0	62.1 ± 4.4	2790	3.6
Kidneys	13.9 ± 1.6	6040	15.0 ± 1.1	6570	8.1	11.9 ± 0.8	5190	−16.4

OLINDA/EXM calculations [2] using representative organ-specific time-activity curve data from clinical patient

^a^113-ml volume

^b^278-ml volume

^c^Changed in absorbed dose compared to using organ calibration factor

## Discussion

### Organ-specific calibration factors

SPECT activity calibration factors measured for 3D printed organ inserts display a clear dependence on organ shape for both ^99*m*^Tc and ^177^Lu, see Fig. 2. For each insert, the ratio of c*f*_*voi*_ to c*f*_*true*_ is constant for ^99*m*^Tc and ^177^Lu, demonstrating that the shape and volume of the VOI are the main influences on relative calibration factor. The measured values of c*f*_*true*_ (Table 2) reflect the gamma emission probabilities for the 140 keV emission from the decay of ^99*m*^Tc and the 208 keV emission following the decay of ^177^Lu (89 and 10.4 %, respectively) and the system sensitivities at these energies.

Figure 2 shows close agreement between the measured VOI calibration factors and theoretical values, as defined in Eq. 7 with a FWHM of 15 mm (*σ*= 6.4 mm), for insert volumes >16 ml and for ^99*m*^Tc and ^177^Lu. The converging value of FWHM (which will vary with the choice of collimator, orbital distance and emission energy) for ^99*m*^Tc (LEHR collimators) and ^177^Lu (MEGP collimators) at a 25-cm orbital radius is consistent with previously reported data [13].

For all insert data, discretization effects will give counts in voxels adjacent to the VOI, analogous to background activity. This effect is negligible for large volume inserts resulting in good agreement with calculated values from Eq. 7. For the smaller inserts, the CT-defined VOIs correspond to a relatively small number of voxels (20–180 voxels in 2–16 ml) and so the effect is enhanced, resulting in the underestimation of the calculated values (see Fig. 2). The inclusion of an additional component, as previously discussed in the case of background activity outside the VOI, is required to fully reproduce the measured values of c*f*_*voi*_/c*f*_*true*_ below 16 ml. Compensation for this effect is particularly important for accurate tumour activity quantification.

This work has demonstrated a clear relationship between measured whole organ calibration factors and VOI shape. The observed variation in calibration factor has been reproduced with a simple voxel level calculation over two orders of magnitude change in insert volume. These results highlight the potential for individualised whole organ activity quantification, corresponding to patient organ VOIs. An alternative method for deriving whole organ calibration factors based on small VOIs placed in areas of homogeneous activity within organs is reported in [14]. It should be noted however that this method does not account for any inhomogeneous activity distribution within an VOI or for the the shape of the VOI, as presented in this paper.

The close agreement between measured VOI calibration factors and theoretical values calculated using Eq. 7 shown in this current work highlights the potential to use theoretical VOI specific calibration factors. It should be noted, however, that activity quantification from SPECT imaging relies on a complex chain of related corrections and calibration [1] determinations. The presence of background activity surrounding a VOI, as seen in clinical situations, will result in variable amounts of spill-in which must be accounted for when applying any SPECT calibration. In addition, changes to the specific SPECT reconstruction, including scatter correction and VOI definition techniques [15], will result in different activity calibration factors. Consequently, whilst the analysis presented in this paper has been constrained to focus on the influence of insert geometry on SPECT calibration, the trends in calibration factor resulting from VOI shapes must be determined for each individual SPECT system and choice of reconstruction parameters. The use of 3D printed inserts provides a cost-effective and efficient way for clinical centres to produce phantom data for non-spherical activity distributions. Access to this data is essential for the validation of whole organ calibration factors (and corrections based on them) and has the potential to improve the accuracy of any SPECT quantification.

### Impact on absorbed dose calculations

The representative absorbed dose calculations for ^177^Lu shown in Table 3 demonstrate a significant variation in calculated dose to all organs with changing calibration factor. The observed variations in organ-specific calibration factors from a spherical factor (as shown in Fig 2) translate directly into a corresponding change in residence time and subsequent absorbed dose calculation.

In particular, a 24 % reduction in calculated absorbed dose for the liver is seen when using calibration factors from a 3D printed organ insert in comparison to a factor from a standard 113-ml spherical insert. It should be noted that the measured value c*f*_*voi*_ for the liver insert is still 20 % smaller than the true camera sensitivity factor c*f*_*true*_. A calibration factor (c*f*_*voi*_/c*f*_*true*_ = 0.86) for a theoretical 1577-ml sphere, matching the volume of the liver insert, can be calculated using Eq. 7. Applying this larger spherical factor reduces the calculated absorbed dose for the liver to 2880 mGy. The calculated dose is still 7 % higher than when an organ-specific factor is applied (see Table 3) showing that this approach does not fully account for the shape of a VOI. This highlights the importance of using appropriate calibration factors which match both the shape and volume of a VOI. For the spleen and kidneys, a 113-ml sphere is a closer approximation to the organ geometry; however, a reduction in organ dose of 11 and 8.1 %, respectively, is observed when using organ-specific calibration factors compared to a 113-ml spherical calibration factor. These results suggest that the accuracy of absorbed dose calculations may be profoundly influenced by using these generic organ models. There is a potential for further improvement by extending the technique presented in this paper to patient-specific CT-based phantoms [16], providing either individualised phantom insert measurements or a general validation for patient-specific calculations. These techniques have the potential to provide fully individualised activity quantification which can be further extended to provide improved sub-organ volume dosimetry. The most efficient clinical implementation of these techniques on an individual patient basis remains to be established.

## Conclusions

This work demonstrates a novel technique which delivers improved whole organ SPECT activity quantification and absorbed dose calculations for MRT. The inserts developed in this work provide unique SPECT imaging data, with activity distributions matching the underlying mathematical models for MRT dosimetry, which can be used to validate all approaches to SPECT activity quantification. Organ-specific SPECT calibration factors for ^99*m*^Tc and ^177^Lu demonstrate a clear dependence on organ shape for both ^99*m*^Tc and ^177^Lu, with a reduction in calculated absorbed dose compared to simple spherical inserts. The results presented in this work are accurately reproduced with Gaussian kernel-based calculations, over two orders of magnitude change in insert volume. These results highlight the potential for individualised whole organ activity quantification, corresponding to patient organ VOIs, to improve accuracy in whole organ dosimetry. The ability to produce MRT phantom inserts, with a reduced boundary effect between regions of activity in the phantom, using commonly available ABS printers allows complex phantom inserts to be made available to all clinical centres. The ability for clinical centres to test SPECT systems with activity distributions which represent the mathematical models underlying MIRD schema dose calculations is a crucial step in establishing validated MRT dosimetry.
